# Primary care clinicians’ perceptions about antibiotic prescribing for acute bronchitis: a qualitative study

**DOI:** 10.1186/s12875-014-0194-5

**Published:** 2014-12-12

**Authors:** Patrick P Dempsey, Alexandra C Businger, Lauren E Whaley, Joshua J Gagne, Jeffrey A Linder

**Affiliations:** Division of General Medicine and Primary Care, Brigham and Women’s Hospital, 1620 Tremont Street, Boston, MA 02120 USA; Bureau of Infectious Diseases, Massachusetts Department of Public Health, Boston, MA USA; Survey and Data Management Core, Dana Farber Cancer Institute, Boston, MA USA; Harvard Medical School, Boston, MA USA

**Keywords:** Bronchitis, Respiratory tract infections, Anti-bacterial agents, Primary health care, Qualitative research

## Abstract

**Background:**

Clinicians prescribe antibiotics to over 65% of adults with acute bronchitis despite guidelines stating that antibiotics are not indicated.

**Methods:**

To identify and understand primary care clinician perceptions about antibiotic prescribing for acute bronchitis, we conducted semi-structured interviews with 13 primary care clinicians in Boston, Massachusetts and used thematic content analysis.

**Results:**

All the participants agreed with guidelines that antibiotics are not indicated for acute bronchitis and felt that clinicians other than themselves were responsible for overprescribing. Barriers to guideline adherence included 6 themes: (1) perceived patient demand, which was the main barrier, although some clinicians perceived a recent decrease; (2) lack of accountability for antibiotic prescribing; (3) saving time and money; (4) other clinicians’ misconceptions about acute bronchitis; (5) diagnostic uncertainty; and (6) clinician dissatisfaction in failing to meet patient expectations. Strategies to decrease inappropriate antibiotic prescribing included 5 themes: (1) patient educational materials; (2) quality reporting; (3) clinical decision support; (4) use of an over-the-counter prescription pad; and (5) pre-visit triage and education by nurses to prevent visits.

**Conclusions:**

Clinicians continued to cite patient demand as the main reason for antibiotic prescribing for acute bronchitis, though some clinicians perceived a recent decrease. Clinicians felt that other clinicians were responsible for inappropriate antibiotic prescribing and that better pre-visit triage by nurses could prevent visits and change patients’ expectations.

**Electronic supplementary material:**

The online version of this article (doi:10.1186/s12875-014-0194-5) contains supplementary material, which is available to authorized users.

## Background

Acute bronchitis is a cough-predominant upper respiratory illness, lasting less than three weeks, in a patient without underlying cardiopulmonary disease [1]. National guidelines and performance measures, based on randomized controlled trials and meta-analyses, recommend avoiding antibiotic prescribing for patients with acute cough/acute bronchitis [2-5]. Over the past several decades, the Centers for Disease Control and Prevention and other organizations have continued efforts to discourage antibiotic prescribing for acute bronchitis [6]. Despite these efforts, physicians in the United States prescribe antibiotics in over 65% of acute cough/acute bronchitis visits [7,8]. Unnecessary antibiotic prescriptions provide no clinical improvement, expose patients to the risk of adverse drug events, increase the prevalence of antibiotic-resistant bacteria, and increase healthcare costs [9].

Prior studies have assessed clinicians’ understanding and attitudes towards antibiotic prescribing for acute bronchitis and other respiratory infections. Most of these studies, summarized in two recent systematic reviews, were performed outside the United States or were published over a decade ago [10,11].

In order to understand contemporary reasons for antibiotic prescribing for acute bronchitis in the United States, clinicians’ familiarity with the clinical guidelines for acute bronchitis, barriers to guideline adherence, and clinicians’ views on potential solutions for unnecessary antibiotic prescribing, we conducted semi-structured, qualitative interviews with primary care clinicians.

## Methods

### Setting and participants

Thirteen primary care clinicians – 12 medical doctors and 1 nurse practitioner – were recruited from 3 Brigham and Women’s Primary Care Practice-Based Research Network-clinics in Boston. The clinics consisted of 1 hospital-based practice and 2 urban community health centers with approximately 175 clinicians which all serve a socioeconomically, racially, and ethnically diverse patient population. Clinicians were recruited via emails sent by study staff to practice medical directors of the 3 clinics who, in turn, forwarded the request to primary care clinicians. If clinicians expressed interest in participating, study staff contacted the clinicians and obtained consent to participate. Interviews took place during March 2011. Clinicians were internal medicine physicians or internal medicine nurse practitioners, 69% female, with a mean age of 43, and an average of 14 years of experience. Clinicians received US$20 for their participation. The Brigham and Women’s Human Research Committee approved the study protocol.

### Interview guide development

The interview guide was developed as part of an iterative process between our team and a consulting Ph.D.-level, qualitative researcher with extensive training and 12 years of research experience in mixed methods design, field methods, and analysis. We used conceptual models of guideline adherence [12] and appropriate antibiotic prescribing [13] to develop the interview guide, ensuring that a range of concepts would be included. The guide focused on the following goals and domains (Appendix 1):

1. Understand clinicians’ contemporary views on acute bronchitis guidelines and antibiotic prescribing;
2. Identify what clinicians felt were the main barriers to antibiotic guideline adherence;
3. Seek clinicians’ ideas and suggestions for methods of improvement for the management of acute bronchitis, clinic workflow, and patient education materials.

### Interview conduct

Interviews were conducted by a medical anthropologist with a Masters Degree in Applied Socio-Cultural Anthropology and 17 years experience in conducting qualitative research interviews. Interviews were completed over the phone and lasted between 30 and 60 minutes. The interviewer obtained IRB-approved verbal informed consent to conduct and record the interview as well as use the content of the interview for analysis and publication. The interviewer generally followed the interview guide, asked open-ended questions, but also improvised questions to elicit additional responses or clarify prior responses. If the participant did not offer solutions for domain 3, the interviewer mentioned specific potential interventions included in the interview guide. We conducted 13 interviews to collect general assessments of attitudes regarding acute bronchitis, reasons for antibiotic prescribing, and solutions to reduce antibiotic prescribing for acute bronchitis. Preliminary analysis of the interviews suggested we were approaching thematic saturation after 12 interviews.

### Data collection and analysis

Each interview was recorded, transcribed verbatim, and analyzed according to a conventional comprehensive qualitative analysis method [14-17]. We used a two-stage coding process: structural coding (Level 1) and thematic coding (Level 2). Structural coding followed the structure of the interview guide: every question received a structural code that was applied to the appropriate text. Thematic coding was based on themes that arose from the structural coding, and was applied in a second-pass analysis. Thematic analysis was inductive and followed the structure of the interview. The thematic content analysis was at the question level, such that themes emerged from the questions. We resolved differences in interpretation through discussion. We used NVivo (Version 8, QSR International) to code, categorize, search, retrieve, attach analytical memos and create conceptual relationship networks in our textual data that had been taxonomically coded. Once the 2-stage coding process was completed and reviewed by the entire research team, we generated a comprehensive thematic analysis summary report, including exemplary quotes. The analysis was performed and the summary report was written and delivered by the same experienced qualitative researcher that completed the interviews.

In this manuscript, we organized themes by decreasing number of participants who discussed each theme. While this provides quantitative impression of the frequency with which participants discussed each theme, we do not mean to imply that themes with a higher number of discussants are necessarily more important.

## Results

Twelve themes emerged from the data within the 3 interview guide domains. Within the first domain, “contemporary views on acute bronchitis guidelines and antibiotic prescribing”, there was only 1 consistent theme. Within the second domain, “barriers to guideline adherence”, there were 6 themes. Within the third domain, “methods to reduce antibiotic prescribing”, there were 5 themes (Table 1).

**Table 1:** **Domains and themes regarding antibiotic prescribing for acute bronchitis**

**Domain**	**Theme**
**Contemporary views on acute bronchitis guidelines and antibiotic prescribing**	1. Antibiotics are not indicated for acute bronchitis, but antibiotics are overprescribed
**Barriers to guideline adherence**	1. Perceived patient demand for antibiotics
	2. Lack of accountability or feedback about prescribing
	3. Time and money
	4. Other clinicians’ misconceptions about acute bronchitis
	5. Diagnostic uncertainty and defensive practice
	6. Clinician dissatisfaction with not meeting patient expectations
**Methods to reduce antibiotic prescribing**	1. Patient handouts and other educational materials
	2. Quality reports
	3. Clinical decision support
	4. Pre-visit triage by nurses
	5. Over-the-counter prescription pad

### Domain 1: Contemporary views on acute bronchitis guidelines and antibiotic prescribing

The consistent theme that emerged regarding guidelines and antibiotic prescribing for acute bronchitis was that all participants agreed with guidelines stating that antibiotics are not indicated for acute bronchitis and clinicians felt like antibiotics for acute bronchitis were overprescribed by other clinicians, but not by themselves. One participant stated the guidelines were,

*“fantastic, ‘cause I think there IS a lot of unnecessary prescribing – for colds, for bronchitis, for viral illnesses. So I think that having a guideline might also be helpful in terms of communicating that to patients, and explaining, you know, ‘Not only do I feel this way, but this is a guideline set up for us…by the people in charge of treating’”.* (AB013-MD)

Well established guidelines allowed clinicians to avoid prescribing antibiotics by having an authoritative norm that states antibiotics are not necessary. Despite the availability of guidelines, clinicians felt that other clinicians overused antibiotics.

### Domain 2: Barriers to guideline adherence

#### Perceived patient demand for antibiotics (12 participants)

All but one participant cited patient demand as a reason for prescribing antibiotics for acute bronchitis. Responses were similar among participants, commonly referring to the clinic visit being the logical next step after the patient has tried everything else:

*“I think the patient’s expectation – by the time they’ve come to you, they feel they’ve tried everything else and they want antibiotics, so that’s a big driver”.* (AB002-MD)

One other participant attributed this to living,

“…*in an instant gratification society, and I think [patients] have the impression that an antibiotic is the thing that’ll clear it up really quickly”.* (AB003-MD)

Another clinician stated,

*“I don’t blame them; I blame us. They’re used to it, so, they feel like…they bother to come in, so we should deliver*”. (AB007-MD)

Clinicians felt their patients come to the clinic as a final option, expecting the clinician to provide a quick solution.

Two clinicians mentioned serving patients from cultures where antibiotics are over-the-counter and frequently used:

“*Most of my patients are Spanish-speaking; most of them are from other cultures. You know, antibiotics are available over the counter, most places that they live. You can get ‘em over the counter, down at the little bodega, down at the corner…People go get it, you can buy ampicillin there. You know, and people are used to taking antibiotics all the time, for everything*”. (AB013-MD)

Differing cultural norms lead clinicians to feel that certain populations have an even greater expectation of receiving antibiotics.

Six clinicians indicated patient demand had decreased over the last 5 years:

“*I think it’s much…lower than it used to be. I think people kind of…get it, at least for colds. I think for bronchitis, they still think a bit differently about bronchitis than they do about colds, and so if we use the word ‘bronchitis,’ they are a bit more set up to expect antibiotics. But, I would say most people are fairly accepting of the fact that these things are caused by viruses, and they don’t respond to antibiotics. So, I would say, you know… a fairly significant majority… are okay with that*”. (AB010-MD)

Clinicians perceived that public desire for antibiotics for acute bronchitis has decreased. Clinicians attributed this to the public developing a better understanding of the nature of viruses and antibiotics, but still associated the diagnosis “bronchitis” with a need for an antibiotic.

#### Lack of accountability or feedback about prescribing (10)

Eight participants stated that there was no accountability, oversight, or feedback for prescribing antibiotics that they were aware of.

“*I don’t think I would NOTICE the difference, really, if I… prescribe antibiotics or not… I don’t have any kind of quality measures…that so far I’ve had to…I haven’t really had anyone say anything about it to me*”. (AB011-MD)

Two participants felt no direct accountability except from their own conscience and their responsibility to the public to not contribute to increases in the prevalence of antibiotic-resistant bacteria. One said,

“*I think we’re accountable to…the public, to not render certain antibiotics … powerless, because we’re contributing to the development of resistant organisms*”. (AB005-MD)

#### Time and money (7)

Seven participants acknowledged financial and time-saving incentives that encourage antibiotic prescribing. One participant said that,

“*if you do it, you can see more patients, because you end the visit quicker instead of having a long discussion, trying to get their buy-in to not prescribe. So, actually, yes, in essence [there are time and financial incentives]; but not in the… we-get-paid-to-use-this-drug-stuff-[way]*”. (AB007-MD)

Participants felt that simply prescribing an antibiotic rather than educating the patient meant they could end each encounter faster, see more patients, and be more financially productive. All clinicians who cited time or money as a reason for antibiotic prescribing said that they, themselves, do not do this.

#### Other clinicians’ misconceptions about acute bronchitis (6)

Clinicians felt some of their colleagues did not understand acute bronchitis or were stuck in the habit of prescribing antibiotics for acute bronchitis. Three participants stated that some doctors either do not know or do not believe that acute bronchitis is viral. One participant felt,

“*many physicians believe that… many of the bronchitises are caused by bacterial infections…especially when the sputum is green, which is not really true”.* (AB002-MD)

Another clinician stated that it is more common amongst,

“*doctors that have been practicing for a long time, it’s sort of what they’ve always done. And so, changing behavior is always harder. It’s what they’ve always done and what they’ve seen their colleagues do, and what their patients have asked them to do. And I think changing those behaviors is very challenging*”. (AB001-MD)

Two participants stated that some clinicians might believe antibiotics are the correct treatment:

“*I expect there might be some physicians who actually believe that it’s helpful. Not… putting myself in that category. But that they believe that it’s the right treatment*”. (AB004-MD)

Lastly, one participant felt some clinicians perceive antibiotics as harmless:

“*I think the other thing that’s probably misinformation on physicians’ part is that I think a lot of… and I’m guilty of this, too. I think we think they’re kind of harmless…. what’s the worst that happens if a person was on a course of antibiotics and, they didn’t need it?*” (AB008-MD)

Participants felt there were three main reasons other clinicians prescribed antibiotics for acute bronchitis: thinking that acute bronchitis is caused by bacteria; the fixed behavior of antibiotic prescribing; and thinking that antibiotic prescribing for acute bronchitis is benign.

#### Diagnostic uncertainty and defensive practice (4)

Four participants stated that diagnostic uncertainty influenced their decision to prescribe antibiotics. One clinician stated,

“*the physician can never be 100% sure it’s not a bacterial infection, so they worry about that. So there’s some clinical uncertainty*”. (AB002-MD)

Another participant elaborated on practicing defensively:

“*The guidelines, even though I think they’re well-known, there’s certainly variation in uptake around the fact that… for those kind of question mark calls, people practice defensively and might want to just…‘be on the safe side, give someone antibiotics,’ even though it’s not clear to me that that’s actually the safer choice to do*”. (AB012-MD)

Participants felt that despite well-established guidelines to avoid antibiotic prescribing for acute bronchitis, there will always be some diagnostic uncertainty and associated risk of undertreating an infection when they do not prescribing antibiotics.

#### Clinician dissatisfaction in not meeting patient expectations (3)

Three participants discussed antibiotic prescribing as a response to clinician dissatisfaction in failing to meet perceived patient expectations. One clinician stated,

“*a person who has…taken off work… come in to urgent care, and then for me to turn ‘em around and say, ‘Keep doin’ what you’re already doing,’ I think in some ways doesn’t feel very satisfying as a physician*”. (AB003-MD)

Another clinician stated,

“*if somebody is sick enough to come in…they’re expecting something*…*I think doctors like to do something. You don’t like to think there is nothing you can do, and there’s nothing you can offer*”. (AB005-MD)

Clinicians felt that once a patient makes the effort to come into the clinic it is unsatisfying to not be able to offer a solution.

### Domain 3: Methods to reduce inappropriate prescribing

#### Patient handouts and other educational materials (13)

All clinicians felt it would be helpful to have educational materials for patients that describe the importance of avoiding unnecessary antibiotic use. The majority of responses stated educational materials “*would be really helpful*” (AB008-MD) and would impact prescribing in a “*huge*” way (AB011-MD). Three clinicians felt educational posters are or would be useful in addition to handouts:

“*I think it would be great… [I have] the one that says, ‘Antibiotics don’t work for colds and flu.’ Got that right next to… where my head is, so people, when they’re looking at me, they see the thing saying, ‘Antibiotics are not for you*’”. (AB009-MD)

Clinicians felt that handouts and posters could make for an efficient, established, official-looking means to educate patients about why antibiotics are not needed for acute bronchitis.

One clinician felt that mass media coverage of the issue would be more useful than handouts:

“*I’m always hesitant to hit…with handouts and pamphlets… I don’t think that people really pay attention to that…they’re already kind of in the office. And if they’re in the office, you can just have the conversation…The only way people read about things related to their health is…they’re picking up… magazines, like…Self, or Cosmo, or Health, or, reading The [Boston] Globe, reading The Metro, so… maybe just putting those kind of informational type pieces, in mass media could help*”. (AB003-MD)

Participants felt that mass media coverage of the issue would be more useful than posters and handouts given in the clinic because they are more attention-getting and have the potential to prevent visits in the first place.

#### Quality reports (10)

Ten clinicians felt quality and feedback reports and reviews would be helpful. One clinician stated,

“*that everyone should get their [upper respiratory infection] dashboard …and they should be compared to all their peers in their clinic…in their system…and that it should be publicly available…to patients and supervisors*”. (AB012-MD)

Two clinicians thought group feedback would be useful:

*“[it would be] good at the clinic, then you don’t sort of demonize somebody, and you get to have the education as a group of clinicians in the clinic*”. (AB008-MD)

One clinician recommended the pharmacy track prescribing:

“*I know the pharmacy people…track what we prescribe…specifically, whether it’s generic or name brand…they track these measures and so…they could track who[is] prescribing….antibiotics…Having evidence, like…the number of times that you prescribed antibiotics; these are the cases where you did it*”. (AB013-MD)

Participants responded that ongoing comparison to their peers and pharmacy tracking could provide ongoing encouragement in lowering antibiotic prescribing rates for acute bronchitis.

#### Clinical decision support (8)

Six participants recommended prompts against antibiotics through clinical decision support within the electronic health record. One specifically mentioned an earlier documentation-based clinical decision support prototype:

“*When we had [an earlier form of electronic clinical decision support], that made it really easy, in my opinion, to be able to…more easily flow through these visits…because it had these prompts…that were a little reminder…not only did it have the reminders, but then it had all the kind of symptomatic treatment stuff that you could just print out with a click of a button, like, give them the cough syrup and the Tylenol and the this and the that and the patient handouts on it…I really liked that form, and I wish it hadn’t gone away*”. (AB012-MD)

Two clinicians recommended that the system have clinicians click an indication which reviews the antibiotic order before being accepted, both comparing it to the way radiology ordering is done. Participants indicated that clinical decision support had been and could be a useful tool to reduce inappropriate antibiotic prescribing for acute bronchitis. Having an established means of reviewing best practices, receiving alternative prescription options, and ready-to-print patient information packets had been useful in the past.

#### Pre-visit triage and education by nurses (6)

Six clinicians suggested having nurses – Registered Nurses or Licensed Practical Nurses – perform pre-visit triage to reduce unnecessary visits. One clinician stated:

“*I think a lot of people who have respiratory infections automatically think they need to see a doctor because they think they need a prescription. And…really good education from the…nurses on triage, I think, could actually…do a good job of not even bothering to bring these people in*”. (AB001-MD)

Another clinician added,

“*there has to be some way of identifying those patients so that you don’t…bring them in to the clinic, because I think that there’s a message in bringing somebody into the clinic that we’re actually going to do something that you couldn’t do at home*”. (AB005-MD)

Clinicians expressed that having nurses perform pre-visit triage would be very useful in reducing inappropriate prescribing. Once a patient has made the trip to the clinic it is far more difficult to convince them to simply continue doing the same things. Clinicians thought review and confirmation by triage nurses that the patient was already taking the right steps and did not need to come into the clinic could reduce visits and antibiotic prescribing.

However, three clinicians recommended caution and a potential unintended consequence of pre-visit triage.

“*You don’t want people to feel like they’re getting prejudged as not sick, because then they’re going to come in even more defensive about BEING sick…I’m not sure I’d want somebody that sort of primed…to think they’re not going to get antibiotics, because they’re going to get more geared up*”. (AB007-MD)

#### Over the counter prescription pad (2)

Over-the-counter prescription pads are official-looking, pre-printed forms on which clinicians can quickly recommend non-antibiotic, non-prescription remedies to patients. Two participants endorsed using an over-the-counter prescription pad.

“*I like having the over-the-counter medication kind of prescription pad… because one nice thing about it is, it shows the different classes of medications. Like, I think people say, “Oh, I just took the cold stuff,” but they don’t really know the difference between a pain reliever and a decongestant, and an antibiotic and kind of understanding, well, ‘Did what you picked out, match what your complaints and symptoms are?*’” (AB003-MD)

The second clinician used the over-the-counter prescription pad to put the encounter,

“*in a positive light. You can spin things anyway, ‘Well, the really good news is, you don’t actually need to take an antibiotic. Your body can fight this off, we can help it, you know, with these ways*’”. (AB005-MD)

The over-the-counter prescription pad provides an established, official-looking means to do something for the patient without having to prescribe antibiotics.

## Discussion

We conducted qualitative, semi-structured interviews of primary care clinicians to learn about clinicians’ understanding about acute bronchitis guidelines, barriers to guideline adherence, and thoughts about interventions to decrease antibiotic prescribing for acute bronchitis.

All clinicians agreed with guidelines that antibiotics are not indicated for acute bronchitis. Clinicians perceived that patients continued to have a high level of demand for antibiotics, which remained the largest perceived driver of antibiotic prescribing for acute cough/acute bronchitis. Clinicians wanted to justify patients’ efforts and feel satisfied that they are meeting patients’ expectations. A novel finding was that clinicians noted less demand for antibiotics and better patient understanding in the past five years.

Clinicians generally viewed the problem of antibiotic prescribing for acute bronchitis and solutions to address the problem as being someone else’s responsibility. Participants felt that other clinicians, especially older clinicians, think antibiotics are harmless. Participants mentioned that better pre-visit triage by nurses to identify patients who do not need to make a visit would save patients money, avoid the inconvenience of an unnecessary visit, and decrease overall antibiotic prescribing. Participants generally did not say that clinicians could do a better job coaching and educating patients themselves.

### Comparison with prior qualitative studies

Prior qualitative studies of clinicians’ attitudes towards antibiotic prescribing for respiratory infections were mainly done in Europe or in the United States over 10 years ago. Two recent systematic reviews identified 45 individual studies that examined clinicians’ views of antibiotic prescribing for respiratory infections [10,11]. Only 3 reviewed studies examined prescribing for respiratory infections in the United States in the past decade [18-20]. Mangione-Smith and colleagues found that physicians incorrectly perceive patient questioning about whether or not they need antibiotics as indicating a desire for antibiotics, when in fact questioning was not associated with patient desire for antibiotics [18]. Ong and colleagues found that physicians correctly identified only 27% of patients who wanted antibiotics, but that physician perception of patient desire for antibiotics was the main driver of antibiotic prescribing [19]. Hart and colleagues found that physicians try to balance their own individual “best practice” with perceived patient satisfaction by using education, negotiation, holding-firm, or giving-in [20].

Not included in the systematic reviews, was one newer qualitative study by Ackerman and colleagues [21]. Ackerman and colleagues, as part of a randomized controlled trial of print and electronic-delivered decision support to encourage judicious antibiotic prescribing, found improved awareness of antibiotic resistance, but clinicians still cited patient expectations, time pressure, and diagnostic uncertainty as barriers to judicious antibiotic prescribing [22].

Some of the many qualitative studies from Europe highlight similar themes from our study and other studies regarding antibiotic prescribing for acute cough. These studies describe non-medical reasons for antibiotic prescribing such as the physician having previously missed a diagnosis of pneumonia in a different patient, patient expectations, and maintaining patient satisfaction [23-26]. European studies describe clinical reasons for prescribing antibiotics such as diagnostic uncertainty, fear of making an error of omission (i.e., not prescribing an antibiotic when it was indicated), and reliance on certain clinical factors like lung findings, fever, shortness of breath, sputum production, or crackles [26,27]. One multinational European study by Brookes-Howell and colleagues highlighted international variations in guidelines, systems to reduce patients’ expectation for antibiotics, and availability of antibiotics without a prescription [25]. Recently, a study by Mustafa and colleagues found that Welsh primary care physicians, rather than directly asking about patient desire for antibiotics, preferred to use a “running commentary” during the physical examination – reviewing findings and implications with the patient as they are examined to reassure, share information, and strategically educate – to set patient expectations that an antibiotic prescription was not necessary without appearing curt or dismissive [18,28,29].

### Limitations

Our qualitative study has several limitations. First, clinicians were academically-affiliated and those who agreed to participate may have a particular interest in judicious antibiotic prescribing or be less likely to prescribe antibiotics. Second, participants may have been constrained by the content of the interview guide or given socially desirable answers. However, to avoid topic constraint or social desirability, the interviewer asked open-ended questions and encouraged participants to speak broadly. Third, our sample size was relatively small. However, the low number of emergent themes suggests we approached thematic saturation. Fourth, many of the themes discussed were about participants’ views on other clinicians’ attitudes and behavior, which may or may not reflect other clinicians’ actual attitudes and behavior.

### Implications for clinical practice and practice improvement

If the patient has an acute cough, is not immunosuppressed, does not have a concomitant alternative diagnosis, has normal vital signs and a normal lung exam, antibiotics are not indicated [1]. Despite clinicians’ perception that patients are only interested in antibiotics, other studies have shown that clinicians overestimate this desire and poorly predict which patients want antibiotics [19,30,31]. Clinicians should be educated that antibiotic prescribing is, at most, marginally associated with patient satisfaction [19,31-33]. Future guidelines should address the role of explicit and perceived patient demand in antibiotic prescribing.

Ongoing education and patient-directed materials such as handouts, posters, and over-the-counter prescription pads may further decrease perceived patient demand for antibiotics [34]. Outside of clinic visits, as suggested by our study, improved quality reports – although prior studies about monitoring and feedback are conflicting [35-37] – and pre-visit interventions should continue to be evaluated to decrease inappropriate antibiotic prescribing for acute bronchitis [38]. Accurate point of care tests, which were not addressed in our study, might be acceptable to clinicians and patients [39,40].

Clinicians should feel secure in their decision that antibiotics are not indicated, should not assume patients want antibiotics, and consider using the “running commentary” method to educate and reassure as they do the physical examination [28,29,41]. Additionally, as our participants pointed out and as previously described, clinicians might consider using a term other than “acute bronchitis”, like a “chest cold”, which is accurate, but less associated with patient expectation for antibiotics [42,43]. Rather than focusing on antibiotics, clinicians should focus on symptomatic treatment and educating patients at the time of the visit about realistic expectations for the duration of cough, which lasts, on average, three weeks [18,44].

## Conclusions

In our qualitative study to understand contemporary attitudes regarding acute bronchitis in the United States, we found that clinicians agreed with antibiotic prescribing guidelines for acute bronchitis, felt that patient demand remained the main driver of antibiotic prescribing, but that demand may have lessened recently. Clinicians discussed a range of solutions to decrease antibiotic prescribing. Many of the solutions discussed were done by someone other than the treating clinician, like nurses doing better pre-visit triage and education, or “behind the scenes” solutions, such as clinical decision support and feedback. However, clinicians will continue to address patients with acute bronchitis and are an integral part of implementing solutions to decrease inappropriate antibiotic prescribing.

## Appendix 1

### Interview guide summary

Questions for Clinicians’ Understanding of Antibiotic Treatment for Acute Cough in Primary Care

### Guidelines for antibiotic prescribing

1. Do you have any insight into why antibiotics are overprescribed for acute bronchitis?
2. What do you think of the guidelines?
3. What has your experience been in following the guidelines? How rigid or flexible do you feel they are?
4. Is there any accountability for following or not following them?
5. Are there any financial incentives for or against prescribing antibiotics?
6. Are there instances in which clinicians bend the guidelines?

### Clinicians’ perception of patient desire and demand for antibiotics

1. What do clinicians perceive as patients’ level of desire for and demand for antibiotics?
2. What steps could clinicians at the clinic level take to reduce the over prescribing of antibiotics?

### Clinic workflow

1. Can you think of ways that the clinic workflow could be changed to reduce the prescribing rate of antibiotics?
  - For example, could changes be made before, during, or after the patient visit?

### Let me offer a couple of other suggestions for you to discuss as potential solutions

1. Clinician decision support
2. Quality reports
3. Patient education

### Review of draft education materials

1. What do you think of Additional file 1?
2. What do you think of Additional file 2?
3. What suggestions do you have for improving Additional file 1?
4. What suggestions do you have for improving Additional file 2?
5. Would it be appropriate to educate patients as part of the counseling about the importance of not taking too many antibiotics?

### Final section

1. How do you feel about having a patient handout to give to patients at the end of a visit that details the symptomatic treatment for cough?

## Additional files

Additional file 1:
**Clinician Handout for Patients.**
Additional file 2:
**The Centers for Disease Control and Prevention Get Smart Owl.**
