# Supplementary material for: Primary care clinicians’ perceptions about antibiotic prescribing for acute bronchitis: a qualitative study
Source: BMC Fam Pract. 2014 Dec 12;15:194. doi: 10.1186/s12875-014-0194-5 (PMC4275949; doi:10.1186/s12875-014-0194-5)
Supplement: Additional file 1: — Clinician Handout for Patients. [file 12875_2014_194_MOESM1_ESM.doc]

**Additional file 1: Clinician Handout for Patients**

# Acute Bronchitis

Your doctor has diagnosed you with acute bronchitis. Acute bronchitis is the same thing as a chest cold. Acute bronchitis is an inflammation of the upper airways in the lung. The main symptom of acute bronchitis is cough. Other symptoms of acute bronchitis can include mild shortness of breath, wheezing, mild fever, fatigue, and muscle aches.

Viruses cause acute bronchitis. These viruses are spread through the air, through contact with people who have a cold, and on surfaces that have been contacted by people with a cold. The cough from acute bronchitis lasts for 2 weeks in half of patients and 3 weeks in a quarter of patients.

**Your doctor has prescribed:**

# Insert prescription medications here

**Your doctor has recommended you use:**

*Insert OTC recommendations here*

If you smoke, you should stop. Smokers get acute bronchitis more frequently and the cough lasts longer. You should drink plenty of fluids and get rest while you are sick. Using vaporizers and humidifiers may help your lungs heal more quickly.

**Antibiotics:** Antibiotics do not help acute bronchitis. Antibiotics only kill bacteria and bacteria do not generally cause acute bronchitis. The Centers for Disease Control and Prevention (CDC) recommends that antibiotics should *not* be prescribed for acute bronchitis, regardless of the duration of cough and regardless if sputum is colored. The green and yellow color in sputum is from your own body and is not an indicator of a bacterial infection. If you use unnecessary antibiotics, you run the risk of an allergic reaction and increase your risk of having an infection later with antibiotic-resistant bacteria.

**You should contact your doctor if:**

- Your cough or wheezing lasts more than 1 month
- You develop a high fever (above 102°F), feel very sick or weak, cough up blood, have trouble breathing with lying down, or foot swelling
