# Supplementary material for: Primary care clinicians’ perceptions about antibiotic prescribing for acute bronchitis: a qualitative study
Source: BMC Fam Pract. 2014 Dec 12;15:194. doi: 10.1186/s12875-014-0194-5 (PMC4275949; doi:10.1186/s12875-014-0194-5)
Supplement: Additional file 2: — The Centers for Disease Control and Prevention Get Smart Owl. [file 12875_2014_194_MOESM2_ESM.doc]

**Additional file 2: The Centers for Disease Control and Prevention Get Smart Owl**

Get Smart. Take a look at this chart to find out which upper respiratory infections are usually caused by viruses — germs that are not killed by antibiotics. Talk with your doctor about ways to feel better when you are sick. Ask what you should look for at home that might mean you are developing another infection for which antibiotics might be appropriate.

| Illness |  |  |  |  | Antibiotic  Needed |
| --- | --- | --- | --- | --- | --- |
|  | Virus |  |  | Bacteria |  |
| Cold | ■✔ | | ■ | | No |
| Flu | ■✔ | | ■ | | No |
| Chest Cold (in otherwise healthy children and adults) | ■✔ | | ■ | | No |
| Sore Throats (except strep) | ■✔ | | ■ | | No |
| Bronchitis (in otherwise healthy children and adults) | ■✔ | | ■ | | No |
| Runny Nose  (with green or yellow mucus) | ■✔ | | ■ | | No |
| Fluid in the Middle Ear  (Otitis media with effusion) | ■✔ | | ■ | | No |
